# Arsenic-Induced Genotoxicity and Genetic Susceptibility to Arsenic-Related Pathologies 

**DOI:** 10.3390/ijerph10041527

**Published:** 2013-04-12

**Authors:** Francesca Faita, Liliana Cori, Fabrizio Bianchi, Maria Grazia Andreassi

**Affiliations:** Institute of Clinical Physiology, CNR, via Moruzzi 1, Pisa 56124, Italy; E-Mails: francesca.faita @ifc.cnr.it (F.F.); liliana.cori@ifc.cnr.it (L.C.); fabrizio.bianchi@ifc.cnr.it (F.B.)

**Keywords:** arsenic, biomarkers, genotoxicity, genetic polymorphisms

## Abstract

The arsenic (As) exposure represents an important problem in many parts of the World. Indeed, it is estimated that over 100 million individuals are exposed to arsenic, mainly through a contamination of groundwaters. Chronic exposure to As is associated with adverse effects on human health such as cancers, cardiovascular diseases, neurological diseases and the rate of morbidity and mortality in populations exposed is alarming. The purpose of this review is to summarize the genotoxic effects of As in the cells as well as to discuss the importance of signaling and repair of arsenic-induced DNA damage. The current knowledge of specific polymorphisms in candidate genes that confer susceptibility to arsenic exposure is also reviewed. We also discuss the perspectives offered by the determination of biological markers of early effect on health, incorporating genetic polymorphisms, with biomarkers for exposure to better evaluate exposure-response clinical relationships as well as to develop novel preventative strategies for arsenic- health effects.

## 1. Introduction

Chronic arsenic exposure is an important problem to human health in many parts of the World. Indeed, over 100 million individuals worldwide are exposed to arsenic, mainly through contamination of groundwaters [1]. Arsenic is a metalloid element and exists in organic and inorganic forms. Inorganic arsenic (iAs) is a class I human carcinogen [2] and is associated with adverse effects dependent on dose, duration and exposure frequency. In particular chronic exposure to iAs is associated to an increased risk of skin, bladder, lung, kidney cancers, as well as cardiovascular and neurological diseases, diabetes and non-malignant respiratory diseases [2]. 

The pathogenic mechanisms of iAs related to development of these pathologies are very complex and likely multifactorial. One of principal mechanisms of arsenic toxicity is the induction of a strong oxidative stress with production of free radicals in cells [3]. Indeed, there is increasing evidence that the induction of reactive oxygen species (ROS) plays a crucial role in arsenic toxicity. Several studies have shown that populations chronically exposed to arsenic have significant oxidative stress that, in turn, induces DNA damage [4,5,6], as well as lipid peroxidation and decreased glutathione levels [7,8]. Moreover, the arsenic-induced ROS generation has been related with alteration of signaling pathways inside the cells and transcription factors regulation, which are two mechanisms that play a crucial role in carcinogenesis [9]. In particular, a very recent study has observed that chronic exposure of humans to low levels of arsenic selectively induces oxidative DNA damage of peripheral blood polymorphonuclear cells, increasing and accelerating apoptosis of these cells [10]. Furthermore, interestingly, prenatal arsenic exposure has been associated with oxidative stress in cord blood and with a reduced thymic function, suggesting subsequent immunosuppression in childhood [11]. The oxidative stress induced by arsenic exposure derives mainly from iAs metabolism. Indeed, it has been proposed that the biotrasformation of iAs generates final and intermediate metabolites exhibiting higher toxicity and reactivity compared to the originally ingested iAs [12,13,14]. In the environment iAs can be found in several oxidation states, *i.e*., as trivalent (iAS^III^ or arsenite) and pentavalent (iAs^V^ or arsenate) species [15]. These forms are differently metabolized by mammals and exhibit distinct grades of toxicity. Particularly, the trivalent form is known to be more toxic than the pentavalent form [16]. 

**Figure 1:**
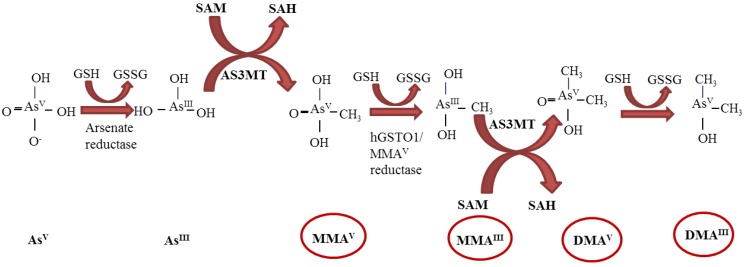
The metabolism pathway of inorganic arsenic showing arsenate reduction to arsenite and methylation to pentavalent and trivalent forms.

In the organism arsenic metabolism may follow two possible pathways: classical reduction and oxidative methylation by the action of arsenic (3^+^ oxidation state) methyltransferase enzyme (ASIIIMT) or a glutathione (GSH) conjugation [1]. In both of these pathways, the end products are monomethylated and dimethylated arsenic metabolites, such as methylarsonic acid (MMA^V^ and MMA^III^ ) and dimethylarsinic acid (DMA^V^ and DMA^III^ ) [1], as shown in Figure 1. Therefore, the oxidative stress induced by chronic exposure to iAs is related to cytotoxic and genotoxic effects in the cells, playing a crucial role in the pathogenesis of diseases, such as diabetes, cardiovascular and nervous systems disorders.

The purpose of this review is to summarize the genotoxic effects of iAs in the cells as well as to discuss the importance of signaling and repair of arsenic-induced DNA damage. The current knowledge of specific polymorphisms in candidate genes that confer susceptibility to arsenic exposure is also reviewed. We also discuss the perspectives offered by the determination of biological markers of early effect on health, incorporating genetic polymorphisms, with biomarkers for exposure to better evaluate exposure-response clinical relationships as well as to develop novel preventative strategies for arsenic- health effects.

## 2. Genotoxicity

The genotoxic role of iAs in the cells has long been controversial. Arsenic is reported to cause DNA modifications such as aneuploidy, micronuclei formation, chromosomal aberrations, deletion mutations, sister chromatid exchange and DNA-protein cross-linking [1]. Several mechanisms have been proposed to explain the genotoxicity of arsenic, as well as induction of oxidative stress and altered patterns of DNA repair [15].

### 2.1. DNA Damage

**Figure 2:**
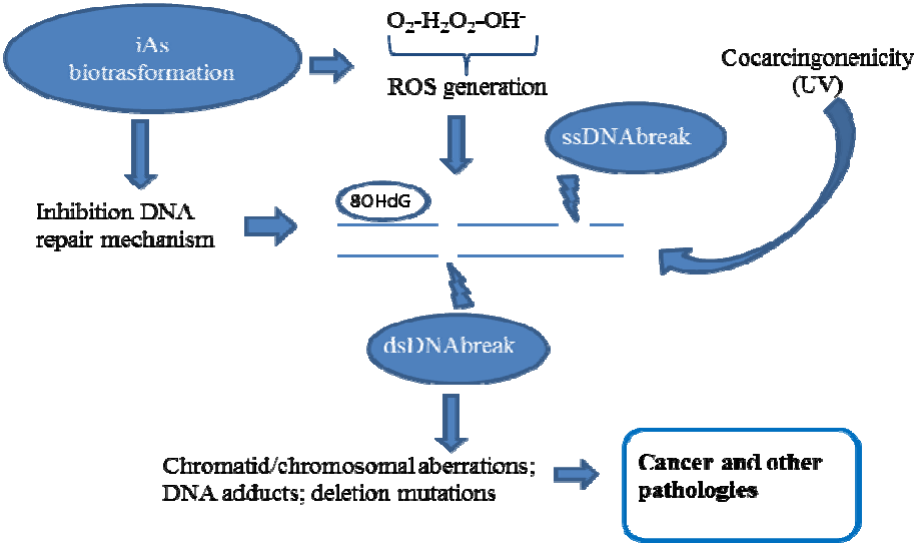
Schematic representation of arsenic genotoxicity.

It has been demonstrated that arsenic does not react directly with DNA [17] and is considered a poor mutagen, as indeed it fails to cause point mutations characteristic of any classical mutagen [1]. However, despite its low capacity to cause mutations, it affects the mutagenicity of other carcinogens (Figure 2). For instance, a synergistic increase in the mutagenic activity of arsenic with UV light has been observed in mammalian and human cells, after exposing the UV-treated cells to arsenic [18,19].

A series of experimental observations suggest that the arsenic genotoxicity is primarily linked to the generation of ROS during its biotransformation [20,21,22]. The ROS production is able to generate DNA adducts; DNA strand breaks, crosslinks and chromosomal aberrations [23,24,25]. The principal mechanism of genetic damage induced by arsenic is via oxidative mechanism (Figure 2).

One of principal effects of oxidative damage to DNA is the DNA base modification. In particular 8-oxoguanine (8-OHdG) is one of most frequently formed DNA nucleobase modifications and is often used in epidemiological studies as a marker for oxidative stress [26,27]. 8-Oxoguanine is a highly mutagenic miscoding lesion that can lead to G:C to T:A trasversion mutations [28]. The presence of oxidative DNA adducts 8-OHdG upon arsenic exposure has been documented in several tissues [29,30].

Recently, urinary 8-OHdG levels were correlated with individual total arsenic level in a human population with low exposure to arsenic and might be indicative of arsenic-induced renal cell carcinoma [31]. Moreover, iAs can induce DNA strand breaks even at low concentrations [15]. Arsenic-induced single-strand breaks are caused either directly by ROS on the DNA bases or indirectly during the course of base excision repair (BER) mechanism [32]. It was observed that human fibroblast cells exposed to iAs exhibit ssDNA breaks and DNA-protein adducts, as well as chromatid exchange [33]. Furthermore, the treatment with O_2_^−^ scavengers and other antioxidants reduce arsenic-induced DNA strand breaks in aortic cells confirming the role of ROS production in the process [34]. In this context it was observed that 1 μM of iAs increases UVR-mediated DNA strand breaks by interfering with Poly-adenosine diphosphate-ribose polymerase 1 (PARP-1) activity, a protein with an important role in the ssDNA and dsDNA break repair process [35].

### 2.2. Chromatid and Chromosomal and Telomere Damage

Arsenic is a known inducer of chromosomal and chromatid aberrations and this involves both clastogenic and aneuploidogenic effects [36]. In recent years several studies have performed cytogenetic monitoring by using chromosomal aberrations assay (CA), micronuclei assay (MN) and Sister Chromatid assay (SCE) in order to detect the genotoxic effects in different population exposed to arsenic, as summarized in the Table 1.

These studies have observed an increase of incidence of CA in individuals with chronic exposure to arsenic [37,38,39,40]. Moreover, it has been observed an increase of frequency of MN in peripheral lymphocytes and in buccal and urothelial cells in exposed individuals in comparisons with a control population. In a study in West Bengal, India, a country with high arsenic contamination, the frequencies of micronuclei in oral mucosal cells, urothelial cells and peripheral lymphocytes were found to be significantly high in exposed participants, compared with unexposed control participants [41]. In Chile Martinez *et al*. have shown a significant increase in the frequencies of MN in peripheral blood lymphocytes of exposed individuals [40]. Furthermore, many studies used buccal epithelial in order to validate this biomarker of genotoxicity demonstrating an increase of MN frequencies in people exposed to As in comparisons with a control population [37,40,42,43,44,45,46,47]. Altogether, these studies showed that chromosomal biomarkers, especially MN and CA, are sensitive biomarkers of early biological effects of iAs exposure [48]. Indeed, only a few studies found weak or no cytogenetic effects in exposed individuals exposed, probably due to the small number of individuals analyzed [49,50,51]. During recent years, large studies have provided consistent evidence that high levels of chromosomal DNA damage in peripheral blood lymphocytes are early predictors of cancer risk and cardiovascular disease [52,53,54]*.*

**Table 1:** Cytogenetic monitoring in populations exposed to arsenic.

Study population, n	Mean arsenic water drinking exposure	Country	Main endpoint result	References
18 exposed subjects 18 controls	1,312 μg/L 16 μg/L	Nevada	1.8 fold increase in bladder cells MN correlation/iAs urinary level	Warner *et al*. [54]
31 exposed subjects 27 controls	408.7 μg/L 29.88 μg/L	Mexico	CA increase in lymphocytes MN increase in oral and urinary cells	Gonsebatt *et al*. [44]
42 exposed subjects 8 controls	410 μg/L <1 μg/L	Finland	CA correlation/urinary As exposure, among current users	Maki-Paakanen [50]
19 exposed subjects 13 controls	527.5 μg/L 4.4 μg/L	USA	3.4 fold increase of MN in buccal cells 2.7 fold increase in bladder cells	Tian [45]
32 cancer cases of risk area 32 controls of risk area	n.d.	Taiwan	No difference in spontaneous and mitomycin C-induced SCE	Liou *et al*. [49]
45 exposed subjects 21 controls	368.11 μg/L 5.49 μg/L	India	MN increase	Basu *et al*. [42]
59 exposed subjects 36 controls	211.70 μg/L 6.35 μg/L	India	CA and SCE increases	Mahata *et al*. [39]
106 exposed subjects 111 controls	>750 μg/L >2 μg/L	Chile	MN increase	Martinez *et al*. [40]
163 exposed subjects 154 controls	214.7 μg/L 9.2 μg/L	India	5.3 fold MN increase in lymphocytes 4.6 fold MN increase in oral cells 4.7 fold MN increase in urothelial cells	Basu *et al*. [41]
45 exposed subjects 25 controls	66.75 μg/L 6.4 μg/L	India	CA and MN increases	Chakraborty *et al*. [43]
422 exposed subjects (244 skin symptomatic) 120 controls	202.33 μg/L 7.16 μg/L	India	CA and MN increases	Ghosh *et al*. [37,38]
200 subjects exposed 165 controls	56.76 μg/L * 117.4 μg/L *	India	MN increase in buccal cells DNA increase in lymphocytes	Vuyyuri [44]
27 exposed subjects 30 controls	>50 μg/L (water drinking) <50 μg/L (water drinking)	Argentina	MN increase in buccal cells	Bartolotta [46]

* Occupational exposure; CA: Chromosomal Aberration; MN: Micronucleus; SCE: Sister Chromatid Exchange; n.d.: not determined.

Moreover, arsenic acts also on expression and length of telomere. In particular, it has been observed that iAs^III^ induce telomerase stimulation at low concentrations, with major effects in female cells respect to male cells. On the contrary, at the concentration of 1 µM, iAS^III^ decrease telomerase expression and telomere length, inducing apoptosis, necrosis and ROS production [55]. Accordingly, a study *in vivo* in a population exposed to arsenic has recently observed that urinary arsenic was positively correlated with the expression of telomerase reverse transcriptase and telomere length [56].

In conclusion, the use of chromosomal biomarkers may be very useful in epidemiological studies in order to provide a better surveillance of arsenic-induced health hazards in populations exposed.

### 2.3. DNA Repair Inhibition

Inhibition of DNA repair processes is considered one of main mechanism of iAs genotoxicity. Nucleotide Excision Repair (NER) and Base Excision Repair (BER) are the processes implicated in the repair of DNA base damage induced by ROS after iAs exposure. In particular the NER mechanism is the major pathway for repairing bulky distortions in DNA double helix, while the BER mechanism is mainly implicated in the repair of single strand breaks induced by RO [57]. Several studies with cultured human fibroblasts showed a reduced DNA repair capacity after iAs exposure [58,59]. Early studies about effect of iAs exposure on DNA repair mechanism proved that arsenic inhibits NER process [58,60,61,62].

Conversely, more recent studies demonstrated that iAs could repress the BER mechanism. [57,63]. Indeed, the BER mechanism is the predominant pathway for DNA lesions caused by ROS and it is possible that it is inhibited by iAs exposure. Sykora *et al*. have observed a dose-dependent down- regulation of mRNA of genes implicated in BER process such as DNA Polymerase beta (Pol beta) and apurinic/apyrimidinic endonuclease (APE1) at doses of As^III^ above 1 μM. However, at lower doses Pol beta mRNA were significantly increased, exhibiting an hormetic effects [64].

Accordingly, a recent study evaluated an arsenic-induced cytotoxic and genotoxic effects under Pol beta deficiency in mouse embryonic fibroblasts. The authors have shown an increased level of DNA damage and significantly delayed DNA damage repair in Pol-beta deficient cells with As exposure, demonstrating an important role for Pol beta in repairing arsenite-induced DNA damage [57]. Moreover, transcription levels of genes related to BER mechanism are altered in lung tissue of mice exposed to iAs in a gene, age, dose and duration manner [65]. Additionally, mRNAs of DNA ligase I and III, implicated in BER mechanisms are significantly reduced in mammalian cells in response to As^III^ [66].

Changes in the BER and NER genes expression levels have been also demonstrated in human exposed populations. Arsenic exposure was associated with decreased expression of the excision repair cross-complementing rodent repair deficiency, complementation group 1 (ERCC1), of the xeroderma pigmentosum group B (XPB) and of the xeroderma pigmentosum group F (XPF) genes in isolated lymphocytes from individuals exposed [59]. A decreased expression of ERCC1 gene at the mRNA and protein levels was also observed among individuals exposed to low dose of arsenic [60].

Recently, Ebert *et al*. have investigated the impact of arsenic on several BER genes in cultured human lung cells, comparing the effects of inorganic arsenite and its trivalent and pentavalent mono and dimethylated metabolites. They have found that arsenite and its metabolites can affect several cellular endpoints related to DNA repair. In particular cellular activity of human 8-oxoguanine DNA glycosylase (hOGG1) was most sensitively affected by DMA^V^; DNA ligase III_α_ LIGIII_α_) protein level by arsenite and X-ray cross complementing protein 1 (XRCC1) by MMA^V^ [63].

## 3. Genetic Susceptibility to Arsenic Toxicity

Recently, epidemiological studies have observed that there is a high inter-individual variability in the susceptibility to arsenic toxicity [1]. This suggests a major role of underlying genetic factors as a cause of this variability. In this regard several studies demonstrated the influence of specific genetic polymorphisms in genes encoding enzymes involved in mechanisms of As metabolism and detoxification, including arsenic(III) methyltransferase (ASIIIMT), glutathione S-transferases (GST) and methylenetetrahydrofolate reductase (MTHFR) enzymes [67,68,69]. Moreover, specific single nucleotide polymorphisms (SNPs) in genes of DNA repair pathways (e.g., hOGG1, APE1, XRCC1, XRCC3 genes) have been shown to reduce the capacity to repair the oxidative damage induced by IAs [70,71] (Table 2).

**Table 2:** Genetic polymorphisms involved in the susceptibility to arsenic toxicity.

Gene symbol	Biological function	SNP	Main associated effect	References
ASIIIMT	As metabolism	G7395A (intronic) T35587C(intronic) G12390C(intronic) C14215T(intronic) Met287Thr A35991G (intronic)	Arsenic metabolite levels	Meza *et al*. [72] Schläwicke Engström *et al*. [69] Hernandez *et al*.[73] Lindberg *et al*. [74] Agusa *et al*. [75] Gong *et al*. [76]
GST-O2	As detoxification	Asn142Asp Ala140Asp	iAs and arsenic metabolites levels Major risk of carotid atherosclerosis	Chung *et al*. [68] Chen *et al*. [77] Hsieh *et al*. [78]
GST-P1	As detoxification	Ile105Val	Arsenic metabolite levels Major risk of TCC Major risk of bladder cancer Major risk of carotid atherosclerosis	Agusa *et al*. [79] Hsu *et al*. [80,81] Lesseur *et al*. [82] Wang *et al*. [83]
GST-M1	As detoxification	Null genotype	iAs and arsenic metabolite levels	Chiou *et al*. [84] Steinmaus *et al*. [85]
GST-T1	As detoxification	null genotype	Arsenic metabolite levels	Chiou *et al*. [86]
MTHFR	As metabolism	Ala222Val	iAs and arsenic metabolite levels	Lindberg *et al*. [74] Schläwicke Engström *et al*. [69]
hOGG1	DNA repair	Ser326 Cys	8-oxoguanine levels	Fujihara *et al*. [70]
APE1	DNA repair	Asp148Glu	8-oxoguanine levels	Fujihara *et al*. [70]
XRCC3	DNA repair	Thr241Met	Arsenic-induced skin lesions; Chromosomal aberrations	Kundu *et al*. [71]
HO1	Inducible antioxidant enzyme	short GT-repeat	BP regulation and cardiovascular mortality risk	Wu *et al*. [87,88]
P53	Tumor suppressor	Arg72Pro	Risk for arsenic-induced kerastosis Risk for renal cell carcinoma	De Chaudhuri *et al*. [89] Huang *et al*. [90]

### 3.1. ASIIIMT Genetic Polymorphisms

ASIIIMT is an S-adenosyl-methionine-dependent enzyme that catalyzes the methylation of arsenite and plays a fundamental role in arsenic metabolism. The human AS3MT gene is approximately 32 kb long and is composed of 11 exons [91]. Several studies have shown that polymorphisms in the AS3MT gene are associated with the efficiency of arsenic biotransformation and cancer risk [67,68]. In 2005, Meza *et al*. showed that three SNPs in introns (G7395A, G12390C and T35587C) were significantly associated with the urinary DMA[V]/MMA[V] ratio in Mexican children [72]. Subsequently, three intronic variant alleles (G12390C, C14215T and A35991G) were found associated with a lower percentage of MMA and a higher percentage of DMA in urine of Argentinians. This study has showed that the presence of these SNPs leads to a higher ratio for second methylation step that converts MMA to DMA [69]. Conversely, the study of SNP A35991G has shown that homozygosity 35991AA was associated with a lower percentage of DMA in urine compared to other genotypes in Vietnamese [75].

Interestingly, different studies have reported that several of these SNPs are in strong linkage-disequilibrium (LD) which also extends to a nearby gene, CYP17A1, forming a large LD cluster in chromosome 10 [73]. Genetic association analysis with As metabolism confirmed a significant association between AS3MT variants in this cluster of linked polymorphisms and arsenic methylation efficiency [73]. Moreover, a recent study has established an association between hypertension, hyperlipidemia, As exposure and SNP A35991G of ASIIIMT gene. In particular hyperlipidemia was associated with genotype AG *vs.* AA of ASIIIMT gene [76].

With regard to genetic variants in the exons, it has been reported that three non-synonymous SNPs (A173W; M287T and T306I) in the ASIIIMT gene significantly alter the levels of enzyme activity and immunoreactive proteins [69]. Among these SNPs the M287T polymorphism is considered to be related to inter-individual variation in the arsenic metabolism [92]. Indeed, in Chile, has been observed that the individuals with one variant allele have a percentage of MMA in urine 8.63 times higher than the individuals with the wild genotype, and that individuals with both variant alleles have twice this MMA increment [93]. A similar result was reported in the central European population [74]. In particular the carriers of the variant allele of the M287T (C➔T) polymorphism had a higher methylation capacity of the enzyme and therefore a higher percentage of MMA metabolite in urine.

These studies showed that the presence of the SNP M287T in gene of ASIIIMT is associated with a higher enzymatic activity of ASIIIMT and alters the standard profile of the urinary metabolites of arsenic, leading to a higher MMA percentage and a higher risk to develop arsenic-related cancers. Moreover, the presence of variant allele of the M287T has been also associated with a major risk to develop carotid atherosclerosis [78].

Accordingly, a recent review summarized genetic studies and findings regarding ASIIIMT SNPs and highlighted that two SNPs (Met287Thr and G12390C) were consistently related to arsenic methylation across diverse populations [94]. Finally, a recent first genome-wide association study (GWAS) of arsenic-related metabolism and toxicity using 30.000 genome-wide SNPs in 1.313 arsenic-exposed Bangladeshi individuals identified genome-wide significant association signals for percentages of MMA and DMA near the AS3MT gene, with five genetic variants showing independent associations [69].

### 3.2. Polymorphisms in GSTs Genes and Other Detoxification Genes

GSTs represent a group of enzymes that are involved in the phase II detoxifications reactions, usually by catalyzing the conjugation of reduced glutathione (GSH) into hydrophobic and electrophilic compounds along with other phase II enzymes.

Many studies have shown that these enzymes are involved in iAs metabolism. In particular four classes of cytosolic GSTs (GST-P1, GST-O1, GST-M1 and GST-T1) have been suggested to take part in iAs biotransformation. In particular several studies have examined the function of two enzymes GST-O1 and GST-O2 which are involved in the reduction of arsenate to arsenite [95,96] by investigating the relationship between GST-O polymorphisms and variation in arsenic methylation. In most studies significant association and, thus, consistent conclusions, have not been found [1,74,97]. However, a recent study in Taiwan showed that the GST-O2 Asn142Asp variant is associated with an increased iAs% [68] and with major risk of arsenic-related bladder cancer [82], while in another study GST-O1 A140D polymorphism is associated with a lower MMA% [77]. Additionally, a marked elevated risk of carotid atherosclerosis was observed in subjects with high arsenic exposure and GSTO risk haplotypes [78].

Furthermore, an increased expression of GST-P1 has been observed in several arsenic-resistant cell lines, suggesting that this enzyme may have an important function in arsenic toxicity [98,99]. In addition, GSTP1 is a polymorphic gene, and the GST-P1 Ile105Val variant influences the geometry of the hydrophobic binding site of GSTP1 enzyme resulting in differences in enzyme specificity and activity [100].

Interestingly, in a Vietnamese population, lower reduction capacity and a lower percentage of urinary As^III^ was observed for Val allele carriers compared to wild-type individuals [79]. Moreover, in another study a gene-environment interaction with this SNP has been observed, showing that individuals homozygous for Val allele with high arsenic exposure had a major risk of bladder cancer [82]. This result is according to two previous studies reporting a major risk of urinary transitional cell carcinoma (TCC) in high arsenic exposed individuals with Val allele compared to wild-type individuals [80,81]. Moreover, Ile105Val variant has been associated with carotid atherosclerosis in population of Taiwan. In particular, a significant joint effect of this variant of GSTP and high arsenic exposure on the risk of developing carotid atherosclerosis was found [83].

Finally, genetic polymorphisms in GST-T1 and GST-M1 has been associated with distinct urinary profiles of arsenic metabolites in populations chronically exposed to iAs [1,86,101]. In particular, a study in Taiwan has shown that individuals with the null genotype of GSTM1 had an increased percentage of inorganic arsenic in urine, whereas those with null genotype of GSTT1 had elevated percentage of DMA in urine [84]. Accordingly, a study showed that Argentinian women with null genotype of GSTM1 had a significantly higher proportion of MMA in urine than women with wild-type genotype [85]. However, other studies didn’t confirm these results: in West Bengal a higher GSTM1 null gene frequencies in asymptomatic individuals than skin symptomatic participants has been observed, and no association was observed for allelic variants in GSTT1 in the two groups [37].

Finally, enzymes involved in the one-carbon metabolism as MTHFR may also indirectly influence the metabolism of arsenic. Recently, the polymorphism A222V of MTHFR gene (associated with reduced enzyme activity) was related with higher %MMA in urine in central European population [74]. Moreover in Argentinian individuals with one or two 222Val allele of MTHFR had lower %DMA and higher %iAs and %MMA, compared with the wild type [1]. In order to confirm the influence of MTHFR in metabolism of arsenic, a very recent study has shown that bladder cancer cases in individuals exposed to As were also 60% less likely to be homozygotes for the A allele in MTHFR gene compared to controls [102].

### 3.3. SNPs in Genes of DNA Repair

Arsenic causes DNA damage and changes the cellular capacity for DNA repair. Therefore, inter-individual variations in DNA repair capacity/efficiency linked to the presence of polymorphisms in DNA repair-related genes may account for different risk of developing damage inducted by arsenic. The base excision repair (BER) pathway is considered an important pathway involved in repair of DNA damage induced by reactive oxygen species. Genetic variants in BER pathway such as hOGG1, XRCC3, XRCC1 and APE1 may modulate and therefore alter the genotoxicity of arsenic. For instance, individuals with hOGG1 326Cys/Cys showed significantly higher urinary 8-OHdG concentrations than did those with 326 Ser/Cys and Ser/Ser in arsenic-exposed Vietnamese, as well for APE1 Asp148Glu, heterozygous subjects as compared to homozygous for Asp/Asp [70]. These results are consistent with previous studies showing lower activity of hOGG1 Cys326 allele [103] and a decreased ability to repair oxidative damage of APE1 variant allele (Glu) [104]. Furthermore, a significant interaction between the genotype and arsenic exposure has been observed for arsenic-related urinary transitional cell carcinoma [80]. Finally, a recent study showed that the variant T241M in XRCC3 gene is associated with a decreased incidence of arsenic-induced skin lesions and a lower level of chromosomal aberrations in individuals exposed to arsenic [71].

### 3.4. Genetic Variants in Other Genes Implicated in Arsenic Susceptibility

Inorganic arsenic has been associated with an increased risk of atherosclerotic vascular disease and cardiovascular mortality [105]. Therefore, many researchers have placed their attention on the study of polymorphisms in genes implicated in the pathogenesis of these diseases. HO-1, a gene involved in adaptive response of the vessel wall to oxidative stress, has been proposed as a protective mode in the progression of atherosclerotic vascular disease [106]. The 5′-flanking region of the gene contains a segment of GT-repeats of varying length; the number of GT repeats have been shown to influence the expression level of the gene [107]. Furthermore, it has been observed that arsenic is a strong inducer of HO-1 expression in cell cultures [108]. Therefore, several studies have shown an association between this genetic polymorphism and hypertension, carotid atherosclerosis and cardiovascular mortality. In particular, it has been observed a protective role of HO-1 short GT-repeat variants in the pathogenesis of these diseases in arsenic exposed populations [87,88].

Finally, several reports have studied the association of polymorphisms in tumor suppressor gene p53 and arsenic-related clinical effects. In West Bengal, the association of three polymorphisms of p53 (codon 72 Arg/Pro; 16-bp duplication at intron 3 and G > A at intron 6) and arsenic-induced kerastosis has been studied, showing that the arginine homozygous genotype at codon 72 and/or homozygous genotype at intron 3 are associated with at increased risk for the development of arsenic-induced kerastosis [89]. Recently, it has been observed that subjects with the p53 codon 72 Pro/Pro genotype had a significantly higher renal cell carcinoma (RCC) risk than those with p53 Arg allele [106]. Moreover, individuals with p53 Arg/Arg genotype had a lower percentage of inorganic arsenic and a significantly higher percentage of DMA, which means a better arsenic methylation capacity [90].

## 4. Conclusions and Future Perspectives

Arsenic has a strong genotoxic potential and is able to cause DNA damage such as aneuploidy; micronuclei formation, chromosomal aberrations, deletion mutations, sister chromatid exchange and DNA-protein cross-linking. Various mechanisms have been hypothesized to explain the cause of this DNA damage, but further studies are needed to establish the mechanism on the basis of the genetic damage induced by arsenic in order to develop specific treatment strategies for the disorders arsenic-related. Furthermore, epidemiological studies have observed that there is an high inter-individual variability in the susceptibility to arsenic-induced toxicity. Several studies have established the influence of genetic polymorphisms on susceptibility to arsenic through their modulation of As metabolism, detoxification and DNA repair. 

Further studies are also required in order to gain a deeper understanding of the health impact of arsenic, and to developing specific therapeutic strategies for the arsenic-related diseases. In general the application of biomarkers in molecular-epidemiological researches constitutes a promising new strategy for enhancing exposure assessment as well as for a better understanding of the mechanisms of action and dose-response relationships for arsenic exposure and human health risk. Indeed, future insights into the arsenic-health risks of require a more accurate characterization of individual exposure and improved knowledge of arsenic-related exposures with early clinical effects. Biomarkers of effect (e.g., methylation changes, chromosomal damage, telomere shortening) indicate early signals of biologic effects preceding disease and/or predict the development and presence of disease. They can also provide individual-based data identifying high-risk groups of overexposed or hypersusceptible subjects. We believe that, in future, the evaluation of the relationship between human exposure to arsenic, estimated using environmental data and assessed through indicators of absorbed dose and biological markers of early effect on health, incorporating genetic polymorphisms will be an important strategy in order to identify identifying at-risk susceptible or resistant subpopulations.

The use of an integrated approach of environmental monitoring and human biomarkers will be able to provide important mechanistic insight into the pathogenesis of disease processes and reduce the time gap exposure and recognition of disease-relevant effects, allowing to develop new and more effective strategies to reduce risk, such as exposure monitoring, health surveillance and individual risk characterization.
